# A novel therapeutic strategy of methicillin-resistant *Staphylococcus aureus*

**DOI:** 10.1016/j.engmic.2025.100231

**Published:** 2025-08-14

**Authors:** Ying Wang, Mengyan Xu, Hanne Ingmer

**Affiliations:** a School of Public Health, Qingdao University, Qingdao, Shandong 266071, China; b Department of Veterinary and Animal Sciences, Faculty of Health and Medical Sciences, University of Copenhagen, Copenhagen 1870, Denmark

**Keywords:** Methicillin-resistant *Staphylococcus aureus*, Antimicrobial resistance, Calcification, Antibody-polysialic acid conjugate, Wall teichoic acid

## Abstract

*Staphylococcus aureus* is a major public health threat, largely due to its remarkable capacity to develop antimicrobial resistance. Zhang *et al.* recently demonstrated a highly innovative approach to eradicate chronic methicillin-resistant *S. aureus* infections by inducing bacterial calcification with antibody-polysialic acid conjugates targeting wall teichoic acids, while simultaneously modulating host immune responses via enhanced calprotectin expression and macrophage activation. Despite limitations, this strategy represents a promising and unconventional therapy to combat resistant *S. aureus* infections.

## Graphical abstract

**Figure:**
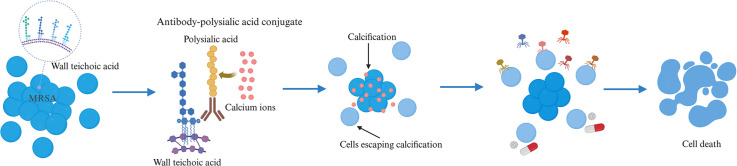

*Staphylococcus aureus* is one of the most serious zoonotic pathogens worldwide, causing a variety of acute and chronic infections, including skin and soft tissue infections (SSTIs), bacteraemia, endocarditis, osteomyelitis, pneumonia, and sepsis. In *S. aureus*, antimicrobial resistance is particularly challenging and estimated to be responsible for approximately 680,000 deaths globally in the single year of 2021 [1]. The most clinically significant challenge is posed by methicillin-resistant *S. aureus* (MRSA) strains, which are resistant to oxacillin and β-lactams [2]. Globally, MRSA was the second leading antibiotic-resistant pathogen in 2019, with the largest increase in both associated and attributed deaths between 1990 and 2021 among 22 interrogated pathogens across 204 countries [1,3]. MRSA infections are often treated with the glycopeptide antibiotic, vancomycin. Although vancomycin-intermediate *S. aureus* strains were reported already in 1996, vancomycin is still the first-line recommended therapeutic agent for MRSA infections [4]. Equally concerning is the spread of specialized *S. aureus* clones in healthcare and community settings, as well as in livestock, with MRSA predominating infections caused by these strain categories [2].

The antibiotic resistance challenges posed by *S. aureus* underscore the urgent need for novel antimicrobial therapies, that ideally are effective against both resistant and susceptible strains, including methicillin-sensitive *S. aureus* (MSSA). Given that resistances in *S. aureus* continuously outpace drug development, and that alternative therapeutic approaches, such as vaccines, remain unfruitful, researchers are increasingly turning to unconventional strategies [5]. Among such therapies is a pioneering approach recently published by Zhang *et al*. in *Nature Biotechnology*, July 2025 [6]. In this study, the authors propose a novel strategy to induce bacterial calcification *in vivo* by applying bacterial-specific antibodies that are coupled to polysialic acid, which attracts calcium ions via its multiple carboxyl groups to encase the pathogen in a calcified shell. Calcification is a natural process that takes particularly in chronic infections. Zhang *et al.* demonstrate that the calcification directed by these antibody-polysialic acid conjugates (APC) specifically targets *S. aureus* wall teichoic acid (WTA) glycopolymer decorated by β-1,4-*N*-acetyl-D-glucosamine (β-1,4-GlcNAc). The resulting calcium deposition on the bacterial surface restricts bacterial growth, alters the expression of multiple bacterial essential genes, including those involved in metabolism, virulence and quorum sensing, and more notably disrupts biofilm formation. Alongside, the calcification elicits the host immune response by boosting the expression of calprotectin subunits S100A8/S100A9 and promoting macrophage activation, consequently facilitating pathogen elimination. The approach was validated in murine models of chronic osteomyelitis and persistent lung infections cause by MRSA, which exhibited considerable therapeutic efficacy and safety.

The epitope recognized by the APC antibody is WTA, a glycopolymer that can be composed of either polyribitol or polyglycerol phosphate units covalently attached to the peptidoglycan layer of the bacterial cell wall. WTAs are a key characteristic of Gram-positive bacteria and exhibit considerable structure diversities in their backbone composition and glycosylation patterns across species and strains. The most common WTA glycosylation in *S. aureus* is the β-1,4-GlcNAc, which is also recognized by the APC conjugates. For the MRSA strain tested in Zhang’s study, 98.8% of cells bound the antibody, whereas in the absence of the corresponding glycosyltransferase (TarS), antibody binding was reduced to 21.8%, similar to a negative control. In contrast, in a mutant lacking the *tarM* gene, which encodes the α-1,4-GlcNAc glycosyltransferase, 100% of the cells bound the antibody. These findings indicate that α-1,4-GlcNAc modification interferes with antibody recognition of the β-1,4-GlcNAc-decorated WTA.

Interestingly, similar observations have been made for bacteriophages, which use WTA as the primary receptor in *S. aureus*. In that context, α-1,4-GlcNAc modification of WTA prevents binding of certain phages, and quorum sensing-mediated regulation of *tarM* expression determines susceptibility to infection by these phages that specifically recognize the β-1,4-GlcNAc-modified WTA [7]. Given the substantial variation in WTA composition among *S. aureus* strains, and the frequent occurrence of infections involving more than one strain of a pathogen, such as mixed infections with both MRSA and MSSA [8,9], the heterogeneity in WTA glycosylation may limit the efficacy of this WTA-targeted antibody therapy. To address this issue, combination therapies incorporating phages that recognize a broad spectrum of WTA modifications may offer a promising solution, which could possibly expand the therapeutic coverage and reduce the likelihood of immune escape.

In addition to strain diversity, there are other limitations of the novel strategy proposed by Zhang and co-authors. First, the study focused exclusively on MRSA in chronic infections using murine models. Chronic infections, such as osteomyelitis and airway infections examined in Zhang’s study, are indeed challenging to treat. However, acute infection cases, including SSTIs, acute pneumonia, and bacteremia, are more prevalent and in urgent need of efficacious cures considering their devastating consequences and substantial public health burden. In our previously reported rural Chinese cohort, most orthopaedic infections originated from *S. aureus*-associated SSTIs, which are relatively common in socioeconomically disadvantaged populations and may progress to osteomyelitis or severe complications, such as amputation, bacteraemia, or even death [10,11]. Further, murine models may have limited value for extrapolating results to humans, because of their different immune responses to *S. aureus* infections [5]. Also mentioned in Zhang’s publication, the high and repetitive dosages of calcium required may not be physiologically compatible or feasible in all tissue compartments, particularly in human patients. Excessive calcium administration could cause hypercalcemia, kidney stone formation, and bone demineralization. Additionally, the potential immunogenicity of APCs, and the need for extensive preclinical validation, such as toxicological and pharmacokinetic studies, all pose translational challenges that must be addressed prior to clinical application. Some of these limitations may be mitigated through combination therapy. It would therefore be of interest to investigate the synergistic effects of APCs when used alongside conventional antibiotics or other therapeutic agents.

Remarkably, the proposed calcification approach achieves both antimicrobial and immunomodulatory functions in one shot, while circumventing a broad range of antimicrobial resistances already developed in bacteria. Furthermore, this approach might complement, rather than compete with, other established treatment options, including antibiotics, phage therapy, and antimicrobial peptides. Even more encouraging is that the calcification platform can potentially be applied to other Gram-positive pathogens and inspire thinking “out-of-the-box” strategies to tackle the constantly growing challenge of antimicrobial resistance.

## CRediT authorship contribution statement

**Ying Wang:** Conceptualization, Writing – original draft, Writing – review & editing. **Mengyan Xu:** Visualization, Writing – review & editing. **Hanne Ingmer:** Conceptualization, Funding acquisition, Writing – original draft, Writing – review & editing.

## Declaration of Competing Interest

The authors declare that they have no known competing financial interests or personal relationships that could have appeared to influence the work reported in this paper.

## References

[1] Antimicrobial Resistance Collaborators (2024). Global burden of bacterial antimicrobial resistance 1990-2021: a systematic analysis with forecasts to 2050. Lancet.

[2] Turner N.A., Sharma-Kuinkel B.K., Maskarinec S.A., Eichenberger E.M., Shah P.P., Carugati M., Holland T.L., Fowler V.G. (2019). Methicillin-resistant Staphylococcus aureus: an overview of basic and clinical research. Nat. Rev. Microbiol..

[3] Antimicrobial Resistance Collaborators (2022). Global burden of bacterial antimicrobial resistance in 2019: a systematic analysis. Lancet.

[4] Liu C., Bayer A., Cosgrove S.E., Daum R.S., Fridkin S.K., Gorwitz R.J., Kaplan S.L., Karchmer A.W., Levine D.P., Murray B.E., Rybak M.J., Talan D.A., Chambers H.F. (2011). Clinical practice guidelines by the infectious diseases society of America for the treatment of methicillin-resistant Staphylococcus aureus infections in adults and children: executive summary. Clin. Infect. Dis..

[5] Clegg J., Soldaini E., Mcloughlin R.M., Rittenhouse S., Bagnoli F., Phogat S. (2021). Staphylococcus aureus vaccine research and development: the past, present and future, including novel therapeutic strategies. Front. Immunol..

[6] Zhang W., Liu L., Zhang Q., Lu H., Li A., Huang Y., Zhang W., Li H., Lu X., Ming X., Yang Z., Shou H., Wang Y., Xia J., Xu F., Wang B. (2025). Inducing bacterial calcification for systematic treatment and immunomodulation against methicillin-resistant Staphylococcus aureus. Nat. Biotechnol..

[7] Yang J., Bowring J.Z., Krusche J., Lehmann E., Bejder B.S., Silva S.F., Bojer M.S., Grunert T., Peschel A., Ingmer H. (2023). Cross-species communication via agr controls phage susceptibility in Staphylococcus aureus. Cell Rep..

[8] Balmer O., Tanner M. (2011). Prevalence and implications of multiple-strain infections. Lancet. Infect. Dis..

[9] Caballero J.D., Wheatley R.M., Kapel N., López-Causapé C., Van der Schalk T., Quinn A., Shaw L.P., Ogunlana L., Recanatini C., Xavier B.B., Timbermont L., Kluytmans J., Ruzin A., Esser M., Malhotra-Kumar S., Oliver A., MacLean R.C. (2023). Mixed strain pathogen populations accelerate the evolution of antibiotic resistance in patients. Nat. Commun..

[10] Wang Y., Liu C., Xia W., Cui Y., Yu L., Zhao D., Guan X., Wang Y., Wang Y., Li Y., Hu J., Liu J. (2024). Association of coagulase-negative staphylococci with orthopedic infections detected by in-house multiplex real-time PCR. Front. Microbiol..

[11] Wang Y., Xia W., Wang Y., Cui Y., Yu L., Liu C., Zhao D., Guan X., Wang Y., Wu S., Li J., Li Y., Hu J., Liu J. (2024). Multiplexed bacterial pathogen detection and clinical characteristics of orthopedic infection in hospitalized patients. Front. Cell. Infect. Microbiol..

